# Deciphering the Contribution of TATA Box and 5′UTR to Defense Signaling in Rice Under Blast Infection

**DOI:** 10.3390/biology14111522

**Published:** 2025-10-30

**Authors:** Xiaoru Fan, Misbah Naz, Yong Zhang, Muhammad Rahil Afzal

**Affiliations:** 1 School of Chemistry and Life Science, Anshan Normal University, Anshan 114007, China; fanxiaoru@asnc.edu.cn; 2 Liaoning Key Laboratory of Development and Utilization for Natural Products Active Molecules, Anshan 114007, China; 3 State Key Laboratory of Green Pesticide, Guizhou University, Guiyang 550025, China; rahil.afzal@yahoo.com; 4 Wuxi Branch of Jiangsu Academy of Agricultural, Wuxi 214000, China; 20210074@jaas.ac.cn

**Keywords:** TATA box, 5′untranslated region, rice cultivars, immune system, gene regulation, pathogen-responsive genes

## Abstract

**Simple Summary:**

Rice blast disease, caused by *Magnaporthe oryzae*, severely threatens global rice production. This study explores how two key DNA regions the TATA box and the 5′ untranslated region (5′UTR) regulate rice defense genes against this pathogen. The TATA box, located in the promoter region, helps start gene transcription by attracting transcription factors, while the 5′UTR controls how efficiently messenger RNA is translated into proteins. Through comparative genomics, gene expression analysis, and mutagenesis, the study reveals that specific TATA box motifs and 5′UTR structures enhance the expression and translation of defense-related genes, thereby strengthening resistance to blast disease. Natural variations in these elements among rice cultivars influence their resistance levels, suggesting that they could serve as valuable molecular markers in breeding programs. Furthermore, modern tools such as *CRISPR/Cas9* can precisely modify these regions to improve disease resistance without affecting crop yield. Overall, the research highlights the TATA box and 5′UTR as vital regulatory elements for fine-tuning rice immune responses and provides a foundation for developing blast-resistant rice varieties through targeted genetic and genomic approaches.

**Abstract:**

The TATA box and 5′untranslated region (5′UTR) are critical regulatory elements that influence gene expression in plant defense responses. In rice (*Oryza sativa*), these elements modulate transcriptional and translational regulation during infection by the blast pathogen *Magnaporthe oryzae*. This study investigates the functional significance of the TATA box and 5′UTR in rice defense signaling by analyzing promoter and 5′UTR variations in key defense-related genes. Through comparative genomics, expression profiling, and mutagenesis assays, we show that 60% of defense genes with specific TATA box motifs exhibit enhanced transcription, while 5′UTR variants increase translational efficiency by up to 2-fold, contributing to blast resistance. These regulatory mechanisms provide a framework for targeted breeding and biotechnological interventions to enhance disease resistance in rice. Our findings highlight the importance of these elements in fine-tuning rice immune responses and suggest potential targets for improving disease resistance in rice cultivars.

## 1. Introduction

Rice (*Oryza sativa*) serves as the primary food source for over half of the world’s population, making its productivity critical for global food security [1]. Rice blast disease, caused by the fungal pathogen *Magnaporthe oryzae*, is responsible for average rice yield losses of about 10% to 30% per year, enough to feed approximately 60 million people. Among the numerous challenges to rice cultivation, blast disease stands out as one of the most destructive, capable of causing severe yield reductions under favorable conditions [2]. The economic and agricultural impacts of blast disease are particularly devastating in developing countries where rice constitutes the dietary staple for millions. Traditional approaches to blast management, including chemical fungicides and cultural practices, have shown limited success and pose environmental concerns [3]. Consequently, developing blast-resistant rice varieties through genetic improvement has emerged as the most sustainable solution, requiring a thorough understanding of the molecular mechanisms underlying rice defense responses against *M. oryzae* [4]. The plant immune system relies on sophisticated gene regulatory networks that enable rapid and precise responses to pathogen invasion. These defense mechanisms involve complex interactions between pathogen-derived molecules and plant recognition systems, triggering cascades of signaling events that culminate in the expression of defense-related genes [5]. The efficiency of these immune responses depends largely on the proper regulation of gene expression at both transcriptional and post-transcriptional levels [6]. Among the key regulatory elements that govern these processes, the TATA box and 5′UTR have emerged as critical components in modulating gene expression during stress responses [7]. While these elements are well-characterized in various biological contexts, their specific roles in rice defense signaling against blast pathogen remain insufficiently explored, representing a significant gap in our understanding of rice immunity [8].

The TATA box, a core promoter element typically located 25–30 base pairs upstream of the transcription start site, plays a fundamental role in transcription initiation by serving as the binding site for the TATA-binding protein (TBP) and associated transcription factors [9]. This element is particularly important for genes that require precise regulation of their expression, including stress-responsive and defense-related genes. In plants, the presence and sequence variation in TATA boxes have been shown to significantly influence the strength and specificity of gene expression in response to biotic stresses [10]. For instance, pathogen-responsive genes in Arabidopsis often contain well-defined TATA boxes that facilitate their rapid induction during infection [11]. Similarly, in rice, many defense-related genes, including those encoding pathogenesis-related (PR) proteins and WRKY transcription factors, possess distinct TATA box motifs that likely contribute to their expression dynamics during blast infection [8]. The functional significance of these motifs is underscored by observations that mutations or natural variations in TATA box sequences can dramatically alter promoter activity, potentially affecting the plant’s ability to mount an effective immune response [12]. Complementing the transcriptional control mediated by the TATA box, the 5′UTR plays a crucial role in post-transcriptional regulation, particularly in determining translational efficiency and mRNA stability [13]. This region, spanning from the transcription start site to the translation initiation codon, contains various regulatory elements that influence protein synthesis, including upstream open reading frames (uORFs), internal ribosome entry sites (IRES), and secondary structures that affect ribosome scanning and recruitment [14]. In the context of plant-pathogen interactions, the 5′UTR serves as a critical checkpoint for translational control, allowing plants to prioritize the synthesis of defense-related proteins while suppressing non-essential cellular processes during infection [15]. Studies in rice have revealed that the 5′UTRs of stress-responsive genes often harbor specific sequence features that enhance translation under pathogen attack, suggesting an evolutionary adaptation to optimize defense responses [16]. Furthermore, some 5′UTRs may interact with RNA-binding proteins or small RNAs to fine-tune gene expression during immune responses, adding another layer of regulatory complexity to the plant defense system [17].

The interaction between rice and *M. oryzae* represents a sophisticated arms race between host defense mechanisms and pathogen virulence strategies. *M. oryzae* is a hemi biotrophic pathogen that initially establishes a biotrophic relationship with the host before transitioning to a necrotrophic phase, causing tissue necrosis and disease symptoms [18]. To counter this threat, rice employs a multi-layered immune system consisting of pattern-triggered immunity (PTI) and effector-triggered immunity (ETI) [19]. PTI is activated when plant pattern recognition receptors (PRRs) detect conserved microbial features known as pathogen-associated molecular patterns (PAMPs), leading to basal defense responses that include cell wall reinforcement, production of antimicrobial compounds, and activation of defense-related genes [20]. When pathogens overcome PTI by secreting effector proteins, ETI is triggered through the recognition of these effectors by resistance (R) proteins, often resulting in a hypersensitive response (HR) characterized by programmed cell death at the infection site [21]. Both PTI and ETI require the coordinated expression of numerous defense genes, the regulation of which involves complex interactions between transcriptional and translational control mechanisms [22]. The importance of precise gene regulation in plant immunity becomes particularly evident when considering the metabolic costs associated with defense responses. Thre constitutive activation of defense mechanisms can lead to significant fitness penalties, including reduced growth and yield [23]. Therefore, plants have evolved sophisticated regulatory systems that allow for the rapid induction of defense genes upon pathogen recognition while maintaining tight control over their expression in the absence of threat [24]. The TATA box and 5′UTR represent two critical components of this regulatory machinery, working in concert to ensure appropriate gene expression patterns during defense responses [25]. Understanding how these elements function in the context of rice blast resistance could provide valuable insights for developing rice varieties with enhanced and durable resistance, potentially through targeted manipulation of these regulatory sequences [6].

Despite considerable progress in understanding rice blast resistance mechanisms, several critical questions remain unanswered regarding the roles of the TATA box and 5′UTR in defense gene regulation [5]. The specific features of TATA box sequences that confer optimal promoter activity during blast infection have not been systematically investigated [5]. Similarly, the contribution of 5′UTR elements to the translational regulation of defense-related mRNAs in rice remains poorly characterized. Addressing these knowledge gaps could reveal novel strategies for improving blast resistance by optimizing the expression of key defense genes through the manipulation of their regulatory sequences [26]. Furthermore, natural variation in these elements among different rice cultivars might explain observed differences in blast resistance, providing potential markers for breeding programs aimed at enhancing disease resistance [27]. The potential applications of this research extend beyond basic science, offering practical solutions for rice improvement [28]. Modern genetic engineering techniques, including *CRISPR*-based genome editing, now allow for precise modifications of regulatory elements such as the TATA box and 5′UTR. By engineering these sequences to optimize defense gene expression, it may be possible to develop rice varieties with enhanced resistance without compromising other agronomic traits [29]. Additionally, identifying natural variants of these regulatory elements associated with blast resistance could facilitate marker-assisted breeding programs, accelerating the development of resistant cultivars. These approaches align with the growing demand for sustainable agricultural practices that reduce reliance on chemical pesticides while maintaining high crop productivity [30].

Research on the regulatory roles of the TATA box and 5′UTR in rice immunity has evolved significantly over the past two decades, with key studies highlighting their importance in defense gene regulation during *M. oryzae* infection [31]. Early work in the 2000s established that many rice defense-related genes, such as PR proteins and WRKY transcription factors, contain canonical TATA boxes in their promoters [32]. These studies demonstrated that mutations in the TATA sequence could reduce promoter activity, impairing the expression of downstream defense genes and increasing susceptibility to blast. For example, transgenic rice lines with modified TATA boxes in the OsPR1b promoter showed weakened induction upon pathogen challenge, confirming the element’s role in transcriptional regulation [33]. The TBP was later identified as a critical mediator of this process and biochemical assays revealed that rice TBP exhibits varying affinity for different TATA variants, with high-affinity binding correlating with stronger defense gene activation [34]. Chromatin immunoprecipitation (ChIP) studies further showed that blast infection triggers rapid recruitment of TBP and RNA polymerase II to TATA-containing defense gene promoters, peaking within 2–4 h post-inoculation. This work underscored the TATA box’s role as a hub for assembling the pre-initiation complex during immune responses [35]. Parallel investigations into the 5′UTR revealed its importance in post-transcriptional regulation. A landmark study by [31] identified uORFs in the 5′UTRs of rice NBS-LRR genes, which encode intracellular immune receptors. These uORFs were shown to suppress translation under normal conditions but permit rapid protein synthesis upon blast infection, likely via stress-induced ribosome bypass mechanisms [36]. Subsequent ribosome profiling experiments confirmed that 5′UTR secondary structures and uORFs dynamically control the translation efficiency of defense mRNAs [37]. For instance, the 5′UTR of OsCERK1, a chitin receptor gene, contains a stem-loop structure that enhances translation during PTI. Comparative genomics studies added an evolutionary dimension to these findings. Genome-wide analyses of resistant (e.g., IR64) and susceptible (e.g., Nipponbare) rice cultivars revealed significant polymorphisms in the TATA boxes and 5′UTRs of defense genes [38,39]. Resistant lines often harbored TATA motifs with higher predicted TBP affinity and 5′UTRs lacking repressive uORFs, indicating positive selection for optimized regulatory elements. Notably, a GWAS study identified a single-nucleotide polymorphism (SNP) in the TATA box of OsPAL6 (a phenylalanine ammonia-lyase gene) that is significantly associated with enhanced blast resistance in indica rice, providing direct evidence that these regulatory variations contribute to the observed phenotypic differences [40]. Despite these advances, gaps remain. Most studies focused on individual genes, leaving the genome-wide distribution of functional TATA/5′UTR variants poorly characterized. Additionally, while in vitro assays dominate TATA-TBP interaction studies, their dynamics in living rice cells during infection remain underexplored. The recent *CRISPR*-editing of TATA boxes and 5′UTRs in rice promises to address these questions, offering new tools to dissect causality between sequence variation and disease phenotypes [41]. In summary, previous studies have demonstrated the functional roles of the TATA box and 5′UTR in regulating rice defense genes against blast disease, primarily through promoter analyses, reporter gene assays, and transcriptional activity studies. These investigations revealed that the presence, position, and sequence context of TATA boxes significantly influence the basal and inducible expression of defense-related genes, such as PR genes and WRKY transcription factors [42]. Similarly, the 5′UTR has been shown to impact mRNA stability, translation initiation, and pathogen-responsive expression, often through the presence of uORFs or secondary RNA structures. The novel aspects of recent research expand beyond traditional promoter studies and delve into epigenetic modifications (such as histone acetylation/methylation and DNA methylation) that modulate chromatin accessibility around these regulatory regions [43]. Additionally, RNA-level regulation, including the involvement of miRNAs, lncRNAs, and tsRNAs, has emerged as a critical mechanism influencing transcript processing and translation via interactions with the 5′UTR. Advances in high-throughput sequencing and transcriptome profiling have also identified alternative transcription start sites and novel 5′UTRs associated with resistance genes [44]. Furthermore, cutting-edge approaches in synthetic biology and promoter engineering are enabling the design of chimeric promoters with optimized TATA boxes and customized 5′UTRs to drive pathogen-inducible and tissue-specific expression of blast resistance genes. Comparative genomic analyses across resistant and susceptible rice cultivars are uncovering natural variations in these regulatory elements, which can serve as molecular markers for resistance breeding [5].

Collectively, these advances highlight the strategic importance of TATA boxes and 5′UTRs as integral components of rice immune regulation and potential targets for genetic improvement against *M. oryzae* [45]. Collectively, these studies establish the TATA box and 5′UTR as central players in rice blast immunity, operating at transcriptional and translational levels to fine-tune defense outputs. Future work integrating single-cell omics and synthetic biology could unlock their full potential for crop improvement [46].

## 2. The TATA Box: A Transcriptional Control Hub in Rice Immunity

Rice plants, like all living organisms, rely on precise gene regulation to mount effective immune responses against invading pathogens. At the heart of this regulatory system lies the TATA box, a crucial DNA sequence element that serves as the foundation for transcription initiation in many defense-related genes [47]. This conserved motif, typically located about 25–30 base pairs upstream of the transcription start site, plays a pivotal role in rice’s ability to respond to the devastating blast pathogen *M. oryzae*. The importance of the TATA box in plant immunity stems from its function as the binding site for the TBP, which nucleates the assembly of the transcription pre-initiation complex [48]. This molecular machinery is essential for the accurate and efficient expression of genes involved in pathogen recognition, signal transduction, and defense compound production. The molecular architecture of the TATA box in rice follows the general eukaryotic consensus sequence TATAWAW (where W is A or T), but exhibits subtle variations that influence its functional properties. These sequence variations are not random; they have evolved to meet the specific demands of rapid, coordinated gene expression during immune responses. When examining promoters of rice defense genes such as those encoding PR proteins or WRKY transcription factors, one often finds well-conserved TATA boxes with specific flanking sequences that enhance their functionality. Here, there are some examples of defense-related genes in rice that contain the TATAWAW motif in their promoter regions. For instance, the promoter of the PR10a gene (pathogenesis-related 10a), which is known to be involved in rice defense responses against *M. oryzae*, includes a TATAWAW variant (TATAAAT). Similarly, the OsWRKY45 gene, a key transcription factor regulating defense signaling, also carries a TATA-like element in its core promoter that conforms to the TATAWAW consensus. These examples have been added to the revised manuscript in the section discussing TATA box variability and its functional relevance. We believe that this addition provides stronger justification and clearer biological context for the presence and importance of TATAWAW variants in rice defense genes [49]. The physical interaction between the TATA box and TBP induces a dramatic bend in the DNA, creating a platform for the sequential recruitment of other general transcription factors and RNA polymerase II. This bending is crucial as it facilitates the unwinding of DNA and the positioning of the transcriptional machinery at the correct start site [50] (Table 1).

**Figure 1:**
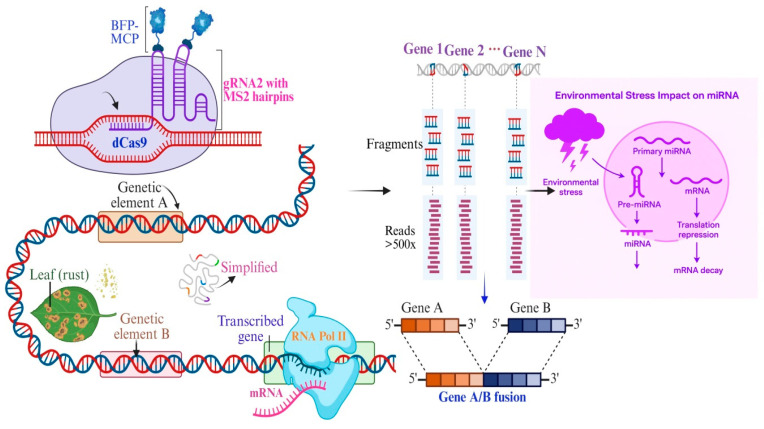
Schematic overview of the experimental strategy to investigate gene fusion events and miRNA regulation under environmental stress. (**Top left**) A *CRISPR-dCas9*-based system is employed using a guide RNA (gRNA2) engineered with MS2 hairpins, which recruits the BFP-MCP complex to a targeted genetic element A. (**Middle left**) Upon environmental stimuli such as leaf rust infection, transcription of downstream gene elements (e.g., element B) is activated by RNA Polymerase II (RNA Pol II), producing mRNA transcripts. (**Bottom left**) Genetic elements A and B are transcribed, leading to potential gene A/B fusion events. Center: Deep sequencing analysis (>500 reads) is performed to identify fusion transcripts among multiple genes (Gene 1 to Gene N). (**Right**) Environmental stress influences miRNA biogenesis and function. Stress conditions affect the processing of primary miRNAs (pri-miRNAs) into precursor (pre-miRNAs) and mature miRNAs, which in turn regulate gene expression through mRNA decay or translational repression. (Bottom right) Illustration of a gene fusion event between Gene A and Gene B, resulting in a chimeric A/B transcript. This integrative experimental approach provides a powerful framework for exploring how environmental stress conditions reshape the transcriptome through both gene fusion events and miRNA-mediated regulation. The use of a *CRISPR-dCas9*-MS2 system allows for precise targeting and visualization of transcriptional dynamics, while deep sequencing enables high-resolution detection of rare fusion transcripts. Importantly, the concurrent modulation of miRNA pathways highlights the multilayered nature of gene regulation under stress, where transcriptional and post-transcriptional mechanisms converge to fine-tune gene expression. These findings not only expand our understanding of plant stress biology but also open new avenues for engineering stress-resilient crops by manipulating gene fusion events or miRNA networks. Further validation of functionally relevant fusion transcripts and miRNA targets will be crucial for translating these insights into practical applications in plant breeding and biotechnology [55].

The dynamics of TBP binding to the TATA box are particularly interesting in the context of rice immunity. Unlike constitutive housekeeping genes that may utilize alternative promoter elements, defense-related genes frequently depend on their TATA boxes for proper induction during pathogen attack [59]. Studies have shown that the affinity of TBP for different TATA variants can vary significantly, with some sequences allowing stronger binding that correlates with more robust transcriptional activation. This becomes critically important during the early stages of blast infection, when the rice plant needs to rapidly upregulate defense genes to contain the invading fungus. The kinetics of this process are remarkable within hours of pathogen recognition, TBP recruitment to defense gene promoters increases dramatically, leading to a corresponding surge in mRNA production (Figure 1) [60]. Beyond the core TATA sequence, the surrounding nucleotides also contribute to regulatory specificity. Certain bases adjacent to the canonical TATA box have been shown to influence the stability of TBP-DNA complexes and the subsequent efficiency of transcription initiation [61]. In rice, these flanking regions often contain binding sites for sequence-specific transcription factors that work in concert with the basal transcriptional machinery to fine-tune defense gene expression. This creates a sophisticated regulatory module where the TATA box serves as both a landing platform for general transcription factors and an integration point for signal-responsive activators [62]. The spatial arrangement of these elements is crucial, as improper spacing between the TATA box and other regulatory motifs can severely compromise immune gene induction [63].

Natural variation in TATA box sequences among rice cultivars provides compelling evidence for their importance in blast resistance. Comparative genomic analyses have revealed correlations between specific TATA variants and disease resistance phenotypes [5]. Some resistant varieties possess TATA boxes with higher affinity for TBP in key defense genes, enabling faster and stronger transcriptional responses upon pathogen detection [8]. These differences, though sometimes involving just single nucleotide changes, can significantly impact the plant’s ability to mount an effective defense. Molecular breeding efforts have begun to exploit this variation, using TATA box characteristics as markers for selecting plants with superior immune response capabilities. The regulation of TATA box accessibility represents another layer of control in rice immunity [64]. Chromatin structure and DNA methylation patterns around TATA elements can determine whether a defense gene remains silent or becomes activated upon infection. In some cases, the TATA boxes of critical immune genes are maintained in an “open “chromatin configuration, poised for rapid activation [65]. Epigenetic preparedness is particularly important for genes that need to respond within minutes to pathogen detection. The blast fungus, in turn, has evolved effector proteins that attempt to interfere with TATA box function, either by blocking TBP access or by recruiting chromatin-modifying enzymes that suppress transcription [66]. This molecular arms race highlights the central importance of the TATA box in the battle between rice and its most destructive pathogen. Experimental manipulation of TATA boxes in rice has provided direct evidence for their role in blast resistance. Reporter gene studies using modified promoters demonstrate that even subtle changes to the TATA sequence can dramatically alter expression patterns during infection [5]. Some engineered variants show improved defense gene induction and correspondingly enhanced resistance, while others lead to weakened responses and increased susceptibility. These findings have important implications for rice improvement strategies, suggesting that the targeted optimization of TATA box elements could be a viable approach to boosting immunity without compromising other agronomic traits [4].

The relationship between TATA box properties and gene expression dynamics during immune responses follows interesting mathematical relationships [67]. Transcriptional output often correlates with TBP binding affinity in a nonlinear fashion, exhibiting threshold effects and saturation kinetics. This nonlinearity helps explain why certain TATA variants are more effective than others in driving defense gene expression during critical phases of pathogen attack. Computational models that incorporate TATA box sequence, TBP binding kinetics, and transcription factor interactions are becoming valuable tools for predicting how genetic variation in these elements might influence rice immunity [68]. Evolutionary analyses of TATA boxes across rice germplasm reveal signatures of selection pressure exerted by blast disease. Certain TATA variants associated with strong defense gene expression appear to be maintained at higher frequencies in regions where blast pressure is intense [69]. This pattern suggests that natural selection has been shaping TATA box sequences to optimize immune responses in different environments. Interestingly, some wild rice relatives harbor TATA variants not found in cultivated varieties, representing a potential reservoir of novel regulatory elements that could be tapped for crop improvement [70].

The study of TATA boxes in rice immunity also provides insights into broader questions about transcriptional regulation in plants. The way rice coordinates the expression of hundreds of defense genes through their promoter elements offers a model for understanding how plants integrate multiple stress signals at the transcriptional level [71]. The TATA box emerges as more than just a passive binding site it functions as an active regulatory node that interprets cellular signals and translates them into appropriate gene expression outputs. This perspective helps explain why mutations in TATA boxes or TBP-related factors can have such profound effects on disease resistance [72]. Looking forward, advances in single-cell technologies and real-time imaging are beginning to reveal how TATA box-mediated transcription varies among different cell types during infection. Early results suggest fascinating spatial and temporal patterns of defense gene activation that correlate with TATA box properties [73]. These findings are refining our understanding of how rice plants allocate defensive resources at the cellular level, with some cells showing much stronger TATA-dependent activation than others. Such heterogeneity may represent an evolved strategy to balance defense costs with the need to limit pathogen spread, The practical applications of this knowledge are already becoming apparent [74]. Molecular breeders are using TATA box characteristics as markers for selecting plants with superior immune response capabilities. Biotechnology approaches are exploring the engineering of synthetic promoters containing optimized TATA elements to drive more effective defense gene expression [75].

The TATA box sequence in rice is generally conserved and does not change directly due to fungal attack, but its transcriptional activity is significantly influenced during infection by pathogens like *M. oryzae* [76]. Fungal invasion triggers complex defense signaling pathways involving salicylic acid, jasmonic acid, ethylene, and reactive oxygen species, which activate defense-related transcription factors such as WRKY, bZIP, and MYB [77]. These transcription factors bind to cis-regulatory elements near the TATA box and modulate RNA polymerase II recruitment, thereby altering the transcription initiation of key defense genes. Furthermore, epigenetic modifications such as histone acetylation and methylation around the TATA box region increase chromatin accessibility, enhancing gene activation in response to stress [63]. In some cases, stress-induced promoter switching or alternative transcription start sites associated with TATA box regions enable a rapid and flexible defense response (Table 2). Thus, although the TATA box sequence remains unchanged, its regulatory function is dynamically modulated by transcriptional and epigenetic mechanisms under fungal stress [78].

### 2.1. The 5′UTR: Gate Keeper of Translational Regulation in Defense Responses

**Figure 2:**
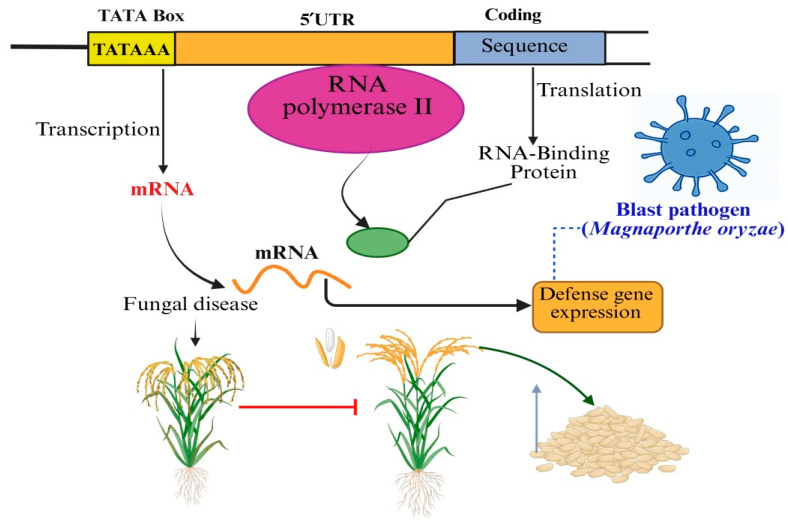
Highlights the integrated regulatory mechanisms at both the transcriptional and translational levels that govern the expression of defense genes in rice during infection by *Magnaporthe oryzae*, the fungus responsible for blast disease.

In the regulation of plant defense, the 5′UTR of mRNA plays a critical role by acting as a molecular gatekeeper that governs translational efficiency and timing (Figure 2) [17]. Positioned upstream of the start codon, the 5′UTR does not encode protein but carries vital regulatory signals that influence how and when a transcript is translated into a functional protein. These regions are particularly significant during biotic stress, where precise and rapid protein synthesis is essential for mounting an effective immune response [84].

At the transcriptional level, the TATA box (depicted in yellow) serves as a crucial cis-regulatory element within the promoter region of defense-related genes. It provides a specific binding site for the transcription machinery, primarily the TATA-binding protein (TBP), which in turn recruits RNA polymerase II to initiate transcription. This results in the synthesis of messenger RNA (mRNA) encoding proteins essential for the plant’s immune response. However, gene expression is not solely controlled at transcription initiation. The 5′ untranslated region (5′UTR) (shown in orange) plays a significant role in post-transcriptional regulation, particularly influencing mRNA stability and the efficiency with which ribosomes translate the mRNA into functional proteins. The interaction of the 5′UTR with RNA-binding proteins can modulate these processes dynamically, allowing the plant to fine-tune protein production in response to changing environmental cues, such as pathogen attack. Upon infection by *M. oryzae*, signal transduction pathways activate transcription factors that bind promoter regions containing the TATA box, enhancing transcription of defense genes [76]. Concurrently, the 5′UTR and associated RNA-binding proteins optimize translation and stabilize the mRNA to ensure sufficient production of defense proteins. These proteins then execute various functions such as strengthening the cell wall, producing antifungal compounds, or signaling further immune responses to mitigate fungal invasion and disease symptoms. The schematic also contrasts this efficient regulatory pathway with a scenario where expression is disrupted (left pathway). In this case, either due to mutations in the TATA box or alterations in the 5′UTR, transcription initiation and/or translation efficiency are compromised. This leads to the insufficient production of defense proteins, resulting in increased susceptibility to blast disease and consequent yield loss, illustrated by the red arrow. Overall, Figure 2 underscores the critical importance of cis-regulatory elements like the TATA box and 5′UTR in orchestrating a coordinated defense response in rice. Their precise sequence and structure ensure that defense genes are expressed at the right time and at adequate levels to protect the plant from pathogen damage, thereby safeguarding crop yield and food security [70].

### 2.2. Structural and Functional Features of 5′UTRs

Structurally, 5′UTRs vary in length and complexity across genes and species, and their functionality is tightly linked to specific motifs and structural features embedded within the sequence [85]. Regulatory elements such as uORFs, internal ribosome entry sites (IRES), and RNA secondary structures like stem-loops and hairpins play key roles in controlling ribosome access and translation initiation [86]. However, under pathogen-induced stress, the repression imposed by uORFs may be lifted, thereby allowing rapid translation of defense-related proteins. Similarly, IRES elements enable cap-independent translation initiation, which is particularly advantageous when the cap-dependent machinery is downregulated during infection. The presence of secondary structures may either hinder or facilitate ribosome scanning and initiation, depending on their positioning and thermodynamic properties [87].

### 2.3. Functional Importance of the 5′UTR in Stress-Response

**Figure 3:**
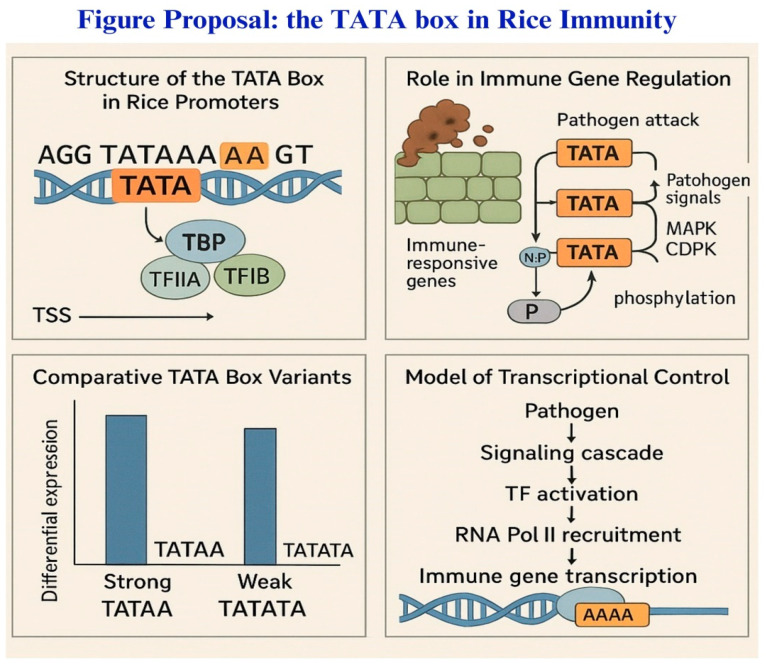
Role of the TATA box in regulating rice immune gene expression in response to pathogen attack. The figure illustrates the structure and function of the TATA box in rice promoters. (**Top left**) The canonical TATA box sequence (TATAAA) is located upstream of the transcription start site (TSS) and is recognized by transcription factors TBP, TFIIA, and TFIIB. (**Top right**) Upon pathogen attack, pathogen signals activate MAPK and CDPK signaling cascades, leading to phosphorylation events that promote transcriptional activation of immune-responsive genes with TATA-containing promoters. (**Bottom left**) Comparative analysis shows differential gene expression associated with strong (TATAA) versus weak (TATATA) TATA box variants. (**Bottom right**) A model of transcriptional control describes how pathogens initiate a signaling cascade, activating transcription factors (TFs) that recruit RNA polymerase II to the promoter, leading to transcription of immune-related genes. The figure highlights the central role of the TATA box in orchestrating defense responses in rice. The coordinated regulation of defense gene expression through key cis-regulatory elements such as the TATA box and 5′UTR is essential for rice to mount an effective immune response against blast disease. Understanding how sequence variations and interactions at these regions influence transcriptional and translational control provides valuable insights for developing disease-resistant rice varieties. Future research focusing on manipulating these regulatory elements could pave the way for innovative breeding strategies aimed at enhancing crop resilience and ensuring sustainable agricultural productivity [40].

In rice, the functional importance of the 5′UTR in stress-responsive translation has been increasingly recognized, especially in the context of resistance against *M. oryzae*, the causal agent of blast disease [88]. Several defense-related genes, R genes and transcription factors involved in immune signaling, exhibit translational control mediated by their 5′UTRs. For instance, the translation of key resistance genes may be selectively enhanced upon pathogen detection, ensuring a swift response without compromising basal growth processes (Figure 3). This stress-responsive translational regulation is vital in managing the energy cost of immunity and allows plants to maintain a delicate balance between defense and development [42].

However, environmental and ecological factors can profoundly influence the activity of key regulatory elements such as the TATA box and the 5′UTR in rice genes involved in defense against the blast pathogen *M. oryzae* [89]. The TATA box, a core promoter element, is essential for accurate transcription initiation by RNA polymerase II. Under changing environmental conditions such as temperature fluctuations, drought, salinity, and pathogen pressure the expression or activity of transcription factors that bind to the TATA box can be altered [90]. These changes may affect the binding affinity or recruitment of the transcriptional machinery, leading to either the enhanced or suppressed transcription of defense genes. Simultaneously, ecological factors such as soil microbiota diversity, plant competition, and co-infection with other pathogens can influence plant hormone signaling pathways (e.g., salicylic acid, jasmonic acid, and ethylene), which in turn regulate transcription factor networks acting on TATA box-containing promoters. In plants, RNA polymerase II binds specifically to promoter regions of genes, guided mainly by DNA sequence motifs such as the TATA box, Inr, or other cis-elements. These sequences enable the recognition by transcription factors and the transcriptional machinery. While the sequence ensures binding specificity, a secondary structure (like chromatin accessibility or DNA looping) plays a crucial role in the regulation of defense gene expression under stress conditions [61]. The 5′UTR, on the other hand, plays a crucial role in post-transcriptional regulation, including mRNA stability, translation efficiency, and localization. Environmental stress can induce changes in the secondary structure of the 5′UTR or promote the use of alternative transcription start sites and uORFs, leading to the altered translation initiation rates of defense proteins. RNA polymerase binds specifically to the promoter regions upstream of genes. This binding is primarily determined by DNA sequence motifs (like the TATA box in eukaryotes or −10/−35 boxes in bacteria). While sequence is essential for specific binding, the secondary structure (e.g., chromatin state or DNA bending) can influence regulation, especially in defense gene activation [91]. The core promoter (including the TATA box and transcription start site) is the primary site for RNA Polymerase II binding, the 5′UTR plays a crucial regulatory role post-transcription initiation, particularly in translation efficiency, mRNA stability, and ribosome recruitment. Studies have shown that both the nucleotide sequence and the secondary structure of the 5′UTR contribute to this regulation. For instance, defense-related genes often contain regulatory motifs or cis-elements within their 5′UTRs (e.g., upstream open reading frames or uORFs) that affect translation. Additionally, RNA secondary structures, such as stem-loops or hairpins, can either facilitate or hinder ribosome scanning and binding, depending on their location and stability and both sequence elements and secondary structures within the 5′UTR contribute to the regulation of defense gene expression, though the exact contribution can be gene- and context-specific. Furthermore, stress-induced RNA-binding proteins and small RNAs may target the 5′UTR, affecting mRNA stability and translation. Together, these regulatory mechanisms allow rice to dynamically modulate gene expression in response to its surrounding environmental and ecological context, enhancing its ability to mount an effective defense against blast disease [92].

Understanding how the TATA box and 5′UTR function as key regulatory elements in rice defense signaling against the blast pathogen *M. oryzae* raises several important scientific questions and possible solutions [93]. One key question is how environmental stresses affect TATA box-binding transcription factors during blast infection. Abiotic factors like drought, salinity, or temperature shifts can alter the expression or activity of these transcription factors, potentially enhancing or weakening their binding to TATA boxes of defense-related genes, thereby modulating transcription initiation [94]. Another question is whether specific sequence features within the 5′UTR of rice resistance genes enhance translation under pathogen attack. Studies suggest that certain motifs, secondary structures, or upstream open uORFs in the 5′UTR can significantly influence translation efficiency, particularly under biotic stress [80].

A related question is whether variations in TATA box or 5′UTR architecture among rice cultivars explain differences in blast resistance. Natural allelic diversity in these regions may lead to differential expression or translation of resistance genes such as Pi genes, OsNPR1, or WRKY transcription factors, and identifying these variants can aid in breeding resistant lines. Another layer of regulation involves epigenetic modifications [95]. Do histone marks or DNA methylation around TATA boxes influence the transcription of defense genes? Emerging evidence suggests that such modifications can affect chromatin accessibility and transcriptional responsiveness during pathogen attack. Furthermore, non-coding RNAs may play a role in regulating 5′UTR activity; for instance, microRNAs or long non-coding RNAs might bind the 5′UTR to suppress or fine-tune translation, adding complexity to post-transcriptional control during immune responses [96]. Addressing these questions requires innovative solutions. Genome editing technologies like *CRISPR/Cas9* can be used to modify TATA box or 5′UTR sequences to improve expression and translation of defense genes specifically under stress conditions. Synthetic promoter engineering offers a strategy to create custom TATA box-containing promoters that are strongly inducible by pathogen presence but remain inactive otherwise, conserving energy. Functional dissection of 5′UTRs using reporter gene assays (e.g., GFP or luciferase fusions) can help identify motifs or structures that enhance translation under infection [46]. Transcriptomic and ribosome profiling under blast stress can reveal transcriptional and translational dynamics of defense-related genes, linking promoter activity and 5′UTR function. Investigating stress-induced epigenetic marks near promoter regions using ChIP-seq can clarify the impact of chromatin remodeling on defense gene activation. In rice, the sequence variation in the TATA box alters transcriptional responsiveness by modulating the binding of the transcription machinery. Meanwhile, 5′UTR variations influence how efficiently the mRNA is translated and stabilized. Together, these elements enable rice plants to dynamically regulate gene expression in response to environmental changes, ensuring survival and adaptation under stress [97]. Finally, exploiting natural diversity by screening traditional or wild rice varieties for beneficial TATA box or 5′UTR polymorphisms, and integrating them into elite cultivars through marker-assisted breeding, can offer durable solutions for improving rice resistance to blast disease in varied environmental and ecological contexts [98].

In natural rice genes, the TATA box and 5′UTR function as vital regulatory elements in orchestrating defense responses against pathogens like *M. oryzae*. The TATA box, a conserved promoter motif located upstream of the transcription start site, serves as the core binding site for general transcription factors, particularly the TATA-binding protein (TBP). This interaction facilitates the recruitment of RNA polymerase II, initiating transcription of defense-related genes such as OsWRKY45, OsNPR1, and OsPR1 [72]. The expression of these genes can be further modulated by stress-responsive transcription factors activated by signaling molecules like salicylic acid and jasmonic acid. Meanwhile, the 5′UTR plays a critical role in post-transcriptional regulation, influencing mRNA stability, translation efficiency, and ribosome binding [99]. Naturally evolved 5′UTRs often contain regulatory features like uORFs, GC-rich regions, and secondary structures, which allow for the context-dependent translation of defense proteins ensuring that energy-intensive responses are tightly regulated and only triggered under pathogenic stress [100].

In contrast, synthetic biology approaches allow for the rational design of TATA boxes and 5′UTRs enhancing the precision and effectiveness of defense gene expression in rice. Synthetic TATA box-containing promoters can be engineered to respond specifically to biotic stress signals, providing high transcriptional activation only during pathogen attack, thereby reducing metabolic costs during normal growth [46]. These promoters can be fused with cis-regulatory elements that are responsive to fungal elicitors or plant hormones, enhancing specificity. Similarly, synthetic 5′UTRs can be customized to eliminate inhibitory features and include translational enhancers or ribosome-binding motifs that increase protein synthesis efficiency under stress conditions. This allows for a more robust and timely production of defense proteins during infection. By integrating these synthetic elements into transgenic or genome-edited rice lines, researchers can achieve enhanced resistance to blast disease with improved regulatory control, opening new avenues for precision breeding and crop protection [101].

### 2.4. Case Studies of 5′UTR-Mediated Defense Gene Regulation in Rice

Case studies in rice have shown that mutations or artificial modifications in 5′UTR can significantly alter the expression of defense genes at the protein level without affecting mRNA abundance, underscoring the translational rather than transcriptional basis of control [102]. For example, manipulating the 5′UTR of resistance gene promoters to remove inhibitory elements or insert pathogen-responsive motifs has been used to boost resistance levels without affecting overall plant vigor [64].

### 2.5. Interaction Between 5′UTRs and RNA-Binding Proteins During Blast Infection

Moreover, the interaction between 5′UTRs and RNA-binding proteins (RBPs) adds another layer of regulation. During blast infection, certain RBPs are recruited to bind specific motifs within 5′UTRs, modulating translation in response to infection signals [103]. These protein RNA interactions may help stabilize the transcript, enhance ribosome loading, or even suppress translation under basal conditions to avoid autoimmune responses. Altogether, the 5′UTR acts as a regulatory hub that translates external stress cues into precise protein production. By integrating structural, sequence specific and protein-interacting features, the 5′UTR enables plants like rice to modulate their defense responses with high temporal and spatial resolution [104]. A deeper understanding of these mechanisms holds promise for designing synthetic regulatory elements that enhance resistance to blast disease and other stresses through targeted translational control [105].

## 3. Multilayered Regulatory Mechanisms

**Figure 4:**
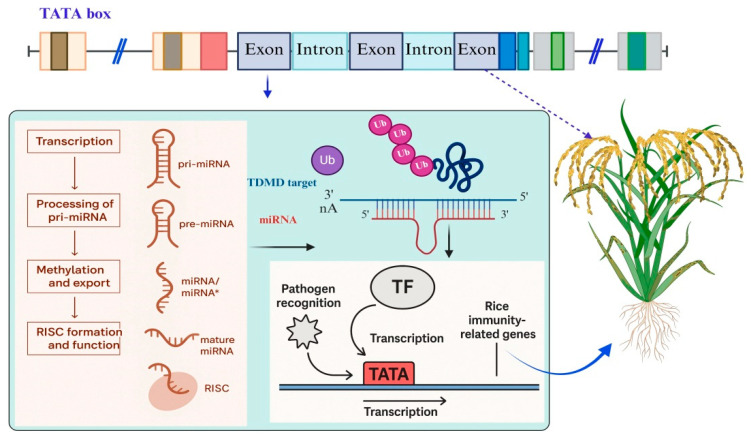
Illustrates the crucial role of the TATA box in regulating immune responses in rice. Top panel: The colored boxes represent structural features of a rice gene. The purple box indicates the TATA box, a core promoter element involved in transcription initiation. Beige boxes represent the 5′ and 3′ untranslated regions (UTRs), while colored boxes (red, blue, green) indicate exons, and light blue connectors indicate introns. These features together define the gene structure involved in transcriptional and post-transcriptional regulation. Bottom left panel: Illustrates the stepwise biogenesis of microRNAs (miRNAs), including transcription of primary miRNA (pri-miRNA), its processing into precursor miRNA (pre-miRNA), methylation, export to the cytoplasm, and formation of the RNA-induced silencing complex (RISC) containing mature miRNAs. Bottom center panel: Depicts transcriptional regulation during pathogen recognition. Pathogen signals activate transcription factors (TFs), which bind to promoters containing the TATA box to initiate transcription of rice immunity-related genes. Top right: A rice plant representing the biological outcome of these regulatory pathways activation of immune-related genes contributes to enhanced disease resistance. When a pathogen such as *Magnaporthe oryzae* invades, it triggers signal transduction pathways that activate specific transcription factors. These transcription factors bind to promoters containing the TATA box, facilitating the recruitment of RNA polymerase II and initiating the transcription of immune-responsive genes. Importantly, variations in the TATA box sequence, such as a strong TATAA motif compared to a weaker TATATA variant, affect how efficiently transcription is initiated. This variation influences the level of gene expression and, consequently, the strength of disease resistance. Therefore, the TATA box serves as a key regulatory element that connects pathogen detection to the activation of defense genes, playing a vital role in rice’s ability to protect itself against pathogens. miRNA* refers to the passenger strand discarded during RISC assembly.

The TATA box and 5′UTR coordinate a multilayered regulatory mechanism that allows rice to mount a dynamic and energy-efficient defense against fungal pathogens (Table 3). While the TATA box enables rapid transcriptional activation of defense genes upon pathogen recognition, the 5′UTR ensures timely and controlled translation of these transcripts into functional proteins [47]. Emerging evidence also suggests that these regulatory regions may be subject to epigenetic modifications or targeted engineering to enhance disease resistance. Understanding their precise roles provides a valuable framework for developing resistant rice varieties through molecular breeding or synthetic biology approaches [106]. Step 1: Pathogen recognition: Rice cells detect *M. oryzae* through pattern recognition receptors (PRRs) on the cell surface. This triggers defense signaling pathways, such as MAPK cascades and the production of defense-related transcription factors (e.g., WRKY, MYB) [107]. Step 2: Activation of promoter region: In response to signaling, transcription factors bind to cis-regulatory elements (e.g., W-box, MYB-box) near the TATA box in the promoters of defense-related genes (e.g., OsPR1a, OsPAL1). The TATA box (~25–35 bp upstream of the TSS) is recognized by TATA-binding protein (TBP), which is part of the TFIID complex [108]. Step 3: Formation of pre-initiation complex (PIC):TBP binding initiates assembly of the pre-initiation complex involving RNA polymerase II and general transcription factors. This enables precise transcription initiation at the TSS, leading to mRNA synthesis of defense genes [109]. Step 4: mRNA processing and 5′UTR function: The transcribed mRNA contains a 5′UTR between the TSS and the start codon. This region may include structural elements, uORFs, or RNA motifs that regulate mRNA stability, nuclear export, and ribosome binding [110]. Step 5: Translational regulation via 5′UTR: Under stress conditions, 5′UTR structures or IRES-like elements help initiate translation even when global protein synthesis is suppressed. If uORFs are present, their translation may be repressed under pathogen stress, allowing ribosomes to access the main coding sequence efficiently [111]. Step 6: Protein synthesis and defense response: Efficient translation of mRNA results in rapid production of defense proteins, including enzymes (PAL, chitinases), PR proteins, and regulatory molecules. These proteins work together to strengthen cell walls, produce antimicrobial compounds, and activate systemic resistance [112]. Step 7: Feedback and fine-tuning: Some defense proteins or RNAs feedback to modulate promoter activity or mRNA turnover, fine-tuning the defense response. TFs regulate defense genes by maintaining low basal expression under normal conditions to conserve energy. Upon stress or pathogen attack, TFs rapidly activate and upregulate these genes for an effective defense response. This fine-tuning ensures precise and timely gene expression for optimal plant survival. While cis-regulatory elements such as TATA box variants and 5′UTR sequences provide the baseline regulatory architecture, it is the trans-acting transcription factors that dynamically interpret these sequences in response to environmental cues [113]. Epigenetic marks may also be added near the promoter or 5′UTR, affecting future responsiveness to pathogen attack (Figure 4) [114].

## 4. Synergistic Roles of TATA Box and 5′UTR in Rice Blast Defense

The defense response in rice against *M. oryzae* requires a tightly coordinated regulatory system that integrates both transcriptional and translational controls [115]. The TATA box, a core promoter element located approximately 25–35 base pairs upstream of the transcription start site, plays a critical role in the precise initiation of transcription by recruiting general transcription factors and RNA polymerase II. Meanwhile, the 5′UTR, situated between the transcription start site and the start codon, fine-tunes gene expression post-transcriptionally by regulating mRNA stability and translation efficiency [116]. Together, these two elements create a regulatory continuum that ensures that immune-related genes are expressed at the right levels, in the right cells, and at the right times during pathogen invasion [19].

### 4.1. Coordinated Transcriptional and Translational Control of Immune Genes

Several key rice defense genes are regulated through the combined action of TATA box and 5′UTR elements [117]. For instance, OsWRKY45, a transcription factor involved in salicylic acid-mediated resistance, contains a well-defined TATA box for transcriptional activation and a structured 5′UTR that influences the translation rate in response to stress [118]. Similarly, Pi-ta and Pi54, two major blast resistance genes, have promoter regions enriched in TATA-like motifs and 5′UTRs that harbor regulatory features such as upstream uORFs and secondary structures that modulate translational readiness. These dual-layer controls provide flexibility and precision, allowing plants to maintain low basal expression of immune genes and rapidly activate them upon pathogen detection [119].

The study reveals a sophisticated dual-layered regulatory mechanism in rice immune genes, where TATA box variants in promoters fine-tune transcription by modulating RNA polymerase II recruitment, as seen in defense genes like PR1, PBZ1, and NAC4, while 5′UTR elements (e.g., uORFs, RNA secondary structures) post-transcriptionally control translation efficiency exemplified by OsWRKY45, where a uORF delays translation until pathogen detection [120]. Under stress, synergistic transcriptional-translational bursts (e.g., OsNPR1, OsCEBiP) amplify immune responses, potentially influenced by phytohormone signaling (e.g., salicylic acid) acting on both TATA-binding proteins and 5′UTR regulators. Evolutionary analyses suggest strong selection for these coupled cis-regulatory elements in rice, with parallels in *Arabidopsis*, hinting at a conserved strategy across plants to balance rapid defense activation with energy efficiency [121].

### 4.2. Examples of Defense Genes Regulated by Both Elements

The synergistic regulatory architecture involving TATA boxes and 5′UTRs is not unique to rice but appears to be evolutionarily conserved across cereal crops such as (wheat, barley, and maize) [122]. Comparative genomic studies reveal that blast resistance orthologs and other R genes in cereals share conserved promoter motifs and 5′UTR structures that point to a shared evolutionary strategy for pathogen defense. This conservation suggests that the integrated transcriptional-translational control of immune genes offers selective advantages in dynamically modulating plant immunity while minimizing fitness costs [123].

### 4.3. Impact of Natural Polymorphisms on Blast Resistance

Natural variation in the TATA box and 5′UTR significantly impacts the level of blast resistance among rice cultivars [124]. Single-nucleotide polymorphisms (SNPs), insertions/deletions (InDels), or epigenetic modifications within these regions can alter promoter strength or disrupt translation-regulating motifs; leading to either enhanced or diminished immune responses [125]. For example, rice landraces with polymorphic TATA motifs upstream of R genes often exhibit differential blast resistance, while variants in the 5′UTR of OsNPR1 have been linked to altered translational efficiency and downstream immune activation. These findings highlight the potential of targeting cis-regulatory elements through breeding or genome editing to fine-tune resistance traits in rice [5,126,127].

## 5. Experimental Approaches to Study TATA Box and 5′UTR Functions

### 5.1. Promoter-Reporter Assays (e.g., GUS, Luciferase)

To elucidate the functional roles of the TATA box and 5′UTR in rice blast resistance, promoter-reporter assays have proven instrumental [128]. In these assays, promoter sequences containing or lacking the TATA box are fused to reporter genes such as β-glucuronidase (GUS) or luciferase and introduced into rice protoplasts or transgenic plants [129]. The activity of these reporters reflects the strength and responsiveness of the promoters under various conditions, including pathogen challenge. Similarly, fusions between 5′UTRs of candidate defense genes and reporter genes allow researchers to quantify how different 5′UTR configurations affect translation efficiency [59]. This strategy has revealed that certain 5′UTR motifs, such as upstream uORFs or secondary structures, can repress or enhance translation under blast-induced stress [130]. Using dual-luciferase systems, side-by-side comparisons can be made between wild-type and mutant regulatory sequences, making this approach a cornerstone in dissecting transcriptional and translational regulation in plant immunity [131].

### 5.2. CRISPR/Cas9-Mediated Mutagenesis of Regulatory Elements

By precisely editing the nucleotide sequences of promoter regions or 5′UTRs, researchers can dissect the functional relevance of specific cis-regulatory motifs in modulating gene expression [45]. Such targeted modifications, enabled by genome editing technologies like *CRISPR/Cas9*, allow for the systematic evaluation of how individual elements such as the TATA box, enhancer sequences, or uORFs influence transcriptional activity, mRNA stability, and translational efficiency [132]. This approach not only elucidates the mechanistic basis of gene regulation but also helps identify key regulatory variants that contribute to enhanced pathogen resistance in crops, thereby informing the development of genetically resilient cultivars [133]. By precisely editing the nucleotide sequences of promoter regions or 5′UTRs, researchers can assess how specific motifs contribute to gene expression and pathogen resistance. Targeted deletions or base substitutions within the TATA box often lead to reduced or abolished transcription initiation, while editing the 5′UTR can disrupt or create regulatory motifs such as IRES elements or uORFs, altering translation without changing mRNA levels [46]. For example, *CRISPR/Cas9* has been used to edit cis-elements upstream of rice blast resistance genes, leading to either enhanced resistance or increased susceptibility depending on the mutation’s impact on gene regulation [134]. This technology not only confirms the functional role of these regulatory sequences but also provides a platform for engineering rice cultivars with fine-tuned immune responses [135].

### 5.3. Ribosome Profiling to Assess Translational Efficiency

Ribosome profiling, or Ribo-seq, is an advanced technique used to monitor translational activity across the transcriptome by capturing ribosome-protected mRNA fragments [136], When applied to rice under pathogen stress, ribosome profiling reveals which transcripts are being actively translated and at what efficiency, allowing for high-resolution mapping of translation start sites and regulatory effects of 5′UTRs [137]. Through this method, researchers have discovered that many defense-related transcripts show selective upregulation at the translational level even when their mRNA levels remain constant. By comparing ribosome occupancy on transcripts with different 5′UTR architectures, the role of translational control elements such as uORFs and RNA secondary structures becomes apparent [138]. Additionally, ribosome profiling can identify shifts in translation efficiency in plants with mutated TATA boxes or edited 5′UTRs, providing direct evidence for the functional interplay between transcriptional and translational regulation [136].

### 5.4. Comparative Genomics of Resistant and Susceptible Rice Lines

Comparative genomics deepens our understanding of the TATA box and 5′UTR functions by enabling the identification of conserved and lineage-specific regulatory elements that contribute to differential gene expression between resistant and susceptible rice lines [131]. Through cross-genotype comparisons, this approach uncovers variations in promoter architecture, including TATA box positioning and sequence conservation, as well as structural and functional diversity in 5′UTRs, which may influence transcription initiation, mRNA stability, and translation efficiency [139]. These regulatory differences can modulate the expression of key stress-responsive or defense-related genes, offering valuable insights into the molecular mechanisms underpinning disease resistance and susceptibility in rice [140]. Genome-wide analyses of natural rice variants have revealed significant sequence variation in promoter and 5′UTR of key immune genes. Resistant cultivars often contain TATA-rich promoters and 5′UTRs with unique regulatory motifs that enhance gene expression under stress, while susceptible varieties may harbor polymorphisms that reduce promoter activity or disrupt efficient translation [141]. Coupling comparative genomics with transcriptomic and ribosome profiling data allows researchers to correlate specific sequence differences with expression patterns and disease outcomes. This integrated approach supports the identification of novel regulatory alleles that can be harnessed in breeding programs or genome editing to develop blast-resistant rice varieties with optimized gene expression profiles [142].

## 6. Biotechnological Applications for Blast Resistance

Biotechnological advances offer promising avenues for enhancing blast resistance in rice through the precise manipulation of cis-regulatory elements such as the TATA box and 5′UTR. Engineering optimized TATA boxes either by strengthening their consensus sequences or by introducing them into naturally TATA-less promoters can significantly boost transcriptional activation of key defense genes, ensuring rapid and robust responses upon pathogen attack [143]. In parallel, synthetic 5′UTRs can be designed to maximize translational efficiency by eliminating inhibitory motifs (e.g., upstream ORFs or stable secondary structures) and incorporating elements that promote ribosome recruitment, thereby enhancing protein output without altering transcription levels. Such synthetic regulatory elements can be integrated into native gene contexts or used in transgenic constructs to fine-tune immune responses. These strategies fall under the broader scope of cis-engineering, which focuses on editing non-coding regulatory regions rather than protein-coding sequences offering the advantage of preserving gene function while modulating expression patterns [144]. Cis-engineering holds substantial potential for accelerating crop improvement programs, especially when combined with genome editing technologies like *CRISPR/Cas9*. However, the deployment of such tools is not without challenges, including the complexity of regulatory networks, the risk of unintended effects on gene expression, and concerns surrounding regulatory approval, biosafety, and public acceptance [145]. Addressing these challenges through rigorous testing, transparent communication, and responsible innovation will be crucial for translating these molecular strategies into sustainable solutions for rice blast management [146].

## 7. Future Perspectives

Despite significant progress in elucidating the roles of the TATA box and 5′UTR in regulating defense responses in rice, several critical questions remain unanswered. The precise mechanisms through which specific nucleotide variations influence transcriptional and translational dynamics during pathogen attack are still poorly understood, particularly in tissue-specific or temporal contexts. Emerging technologies such as single-cell omics offer unprecedented resolution to dissect the spatiotemporal expression patterns of immune genes, while artificial intelligence (AI)-driven promoter and 5′UTR design tools are beginning to enable the predictive modeling of regulatory element functionality. Expanding this research to other major cereal crops such as wheat, maize, and barley will help determine the evolutionary conservation and functional diversity of these elements, offering new targets for breeding broadly resistant cultivars. Integrating regulatory element biology with systems biology approaches including transcriptomics, translatomics, epigenomics, and interactome mapping will provide a holistic understanding of the regulatory networks underpinning plant immunity.

## 8. Conclusions

This review highlights the synergistic roles of TATA boxes and 5′UTRs in regulating gene expression at various levels during rice blast infection. Their coordinated function presents significant biotechnological potential for improving disease resistance. Deepening our understanding of these cis-regulatory elements not only enhances our grasp of plant-pathogen interactions but also opens new avenues for developing durable resistance strategies, supporting more sustainable crop improvement efforts in response to global agricultural challenges.

Furthermore, integrating this knowledge with genome-editing tools, such as *CRISPR/Cas* systems, holds promise for designing promoter architectures and translational control elements that drive durable, inducible, and energy-efficient resistance traits. Ultimately, this line of research supports the development of next-generation crop varieties that are not only disease-resilient but also better adapted to the dynamic challenges of modern agriculture.

## Figures and Tables

**Table 1:** Regulatory roles of TATA Box and 5′UTR in rice defense signaling.

Cis-Element	Core Motif	Transcriptional Regulator	Biological Function in Rice Defense Against Blast Pathogen	Reference
TATA box	TATA(A/T)A(A/T)	TATA-binding protein (TBP), TFIID complex	Initiates transcription by recruiting RNA polymerase II; essential for activating defense gene promoters (e.g., *PR*, *WRKY*, *NBS-LRR* genes) during pathogen attack.	[35]
5′UTR	Variable, includes upstream open reading frames (uORFs), secondary structures	RNA-binding proteins, translational regulators	Modulates mRNA translation efficiency and stability; influences timing and strength of defense gene expression in response to pathogen-induced signals (e.g., salicylic acid, jasmonic acid).	[51]
5′UTR (uORFs)	Upstream open reading frames	Translational machinery components	uORFs in 5′UTRs can regulate downstream gene expression post-transcriptionally, affecting defense responses.	[52]
5′UTR (miRNA targets)	miRNA binding sites	miRNAs (e.g., miR7695)	miRNAs can bind to 5′UTRs, modulating translation of defense-related genes.	[53]
5′UTR (synthetic enhancers)	Synthetic sequences (e.g., AMVE)	Engineered regulatory elements	Insertion of synthetic enhancers into 5′UTRs can boost translation of defense genes.	[54]
5′UTR (AUS modifications)	ATG upstream sequences	Genome editing tools (e.g., *CRISPR*)	Modifying immediate upstream sequences of start codons can influence translation efficiency of defense genes.	[55]
5′UTR (lncRNA interactions)	Long non-coding RNA binding sites	lncRNAs	lncRNAs interacting with 5′UTRs can affect stability and translation of defense-related mRNAs.	[56]
5′UTR (alternative polyadenylation)	Polyadenylation signals	Polyadenylation machinery	Alternative polyadenylation in 5′UTRs can lead to transcript variants affecting defense gene expression.	[57]
5′UTR (miRNA-mediated regulation)	miRNA binding sites	miRNAs (e.g., miR393b)	miRNAs can regulate secretion pathways by targeting 5′UTRs, influencing defense protein exocytosis.	[31]
5′UTR (sRNA networks)	Small RNA binding sites	sRNAs	sRNAs can modulate gene networks by interacting with 5′UTRs, affecting defense responses.	[58]

**Table 2:** Key studies exploring regulatory elements and gene editing for rice blast resistance.

Target Gene(s)/TF	Regulatory Element or Mechanism	Key Findings	Approach Used	Reference
*OsPR1 family*	Promoter and TFBS (TATA box, W-box, GCC-box)	Identified TF binding motifs in *OsPR1* promoters linked to blast response	In silico promoter and TFBS analysis + expression profiling	[72]
*OsBRW1*, *OsSRFP1*	NBS-LRR gene regulation	*OsBRW1* interacts with *OsSRFP1* to regulate immunity and growth balance	Functional genomics + protein interaction assays	[79]
*Multiple resistance genes*	Promoter, TFs, genome editing targets	Reviews precision genome editing for blast resistance	*CRISPR-Cas* toolbox review	[46]
*CYP76M7*	Promoter (early inducible)	Identified promoter activated by *M. oryzae* infection	Cloning + promoter::GUS assay	[80]
*Multiple crop genes*	Gene expression regulation via editing	Overview of genome editing regulating gene expression in crops incl. rice	Review (*CRISPR*, base editing, epigenome editing)	[81]
*OsbZIP1*	bZIP TF gene	Novel bZIP TF induced by *M. grisea*, likely regulating defense genes	Expression analysis + TF identification	[82]
*OsNAC19*	NAC TF gene	*OsNAC19* expression induced upon blast infection	Expression profiling + gene characterization	[83]

**Table 3:** Multilayered regulatory mechanisms coordinated by the TATA box and 5′UTR in rice defense against fungal pathogens.

Regulatory Layer	Component	Function	Role in Fungal Defense	Hormonal Integration	References
Transcription Initiation	TATA box	Core promoter element; recruits RNA polymerase II and general TFs	Enables rapid and accurate transcription of pathogenesis-related (PR) genes	Coordinates with SA/JA-induced TFs (e.g., WRKYs)	[72,82]
TF Binding and Specificity	W-box, TCA-motif, ABRE	Binding sites for WRKY, TGA, and bZIP TFs	Drives defense gene activation in response to *Magnaporthe oryzae*	SA and ABA responsive; WRKY-TGA cross-talk	[46,83]
Translational Regulation	5′UTR uORFs	Modulate translation efficiency and ribosome scanning	Fine-tunes protein production during infection stress	ABA/SA modulated translational buffering	[79,81]
mRNA Stability and Localization	GC-rich motifs, hairpin loops in 5′UTR	Affect mRNA stability and translatability	Ensures sustained expression of defense proteins under biotic stress	JA/ABA signals influence mRNA decay/stabilization	[80]
Energy Efficiency	Combined promoter and 5′UTR elements	Orchestrate transcriptional and translational economy	Prevents overexpression, allowing timely, cost-efficient defense	Feedback regulation via SA-JA antagonism	[81]
Signal Amplification	Promoter-5′UTR synergy	Multiplexed regulation via cis-element clusters	Rapid upregulation of secondary metabolism and PR proteins	Hormonal signal amplification through TF cascades	[83]

## Data Availability

No datasets were generated during the current study.
